# Multi-Spheres Adsorptive Microextraction (MSAμE)—Application of a Novel Analytical Approach for Monitoring Chemical Anthropogenic Markers in Environmental Water Matrices

**DOI:** 10.3390/molecules24050931

**Published:** 2019-03-07

**Authors:** Ana R. M. Silva, Nuno R. Neng, José M. F. Nogueira

**Affiliations:** Centro de Química e Bioquímica, Centro de Química Estrutural, Faculdade de Ciências, Universidade de Lisboa, Campo Grande, Ed. C8, 1749-016 Lisbon, Portugal; rita.mendao@ipleiria.pt

**Keywords:** sorbent-based techniques, multi-spheres adsorptive micro-extraction (MSAμE), floating sampling technology, caffeine and acetaminophen tracers, environmental water matrices

## Abstract

Multi-spheres adsorptive microextraction using powdered activated carbons (ACs) was studied as a novel enrichment approach, followed by liquid desorption and high-performance liquid chromatography with diode array detection (MSAµE(AC)-LD/HPLC-DAD) to monitor caffeine (CAF) and acetaminophen (ACF) traces in environmental matrices. In this study, commercial activated carbons (N, N_OX_, and R) were tested, with the latter showing a much better performance for the analysis of both anthropogenic drugs. The main parameters affecting the efficiency of the proposed methodology are fully discussed using commercial AC(R). Textural and surface chemistry properties of the ACs sample were correlated with the analytical results. Assays performed on 30 mL of water samples spiked at 10 µg L^−1^ under optimized experimental conditions, yielding recoveries of 75.3% for ACF and 82.6% for CAF. The methodology also showed excellent linear dynamic ranges for both drugs with determination coefficients higher than 0.9976, limits of detection and quantification of 0.8–1.2 µg L^−1^ and 2.8–4.0 µg L^−1^, respectively, and suitable precision (RSD < 13.8%). By using the standard addition method, the application of the present method to environmental matrices, including superficial, sea, and wastewater samples, allowed very good performance at the trace level. The proposed methodology proved to be a feasible alternative for polar compound analysis, showing to be easy to implement, reliable, and sensitive, with the possibility to reuse and store the analytical devices loaded with the target compounds for later analysis.

## 1. Introduction

Pharmaceuticals and personal care products (PPCPs) have generated one of the most important emerging environmental issues, particularly in aquatic systems [1]. Wastewaters from municipal treatment plants, livestock farms, veterinary drug use, and residues from hospitals and pharmaceutical manufactures are the best known sources of pollutants discharged into the water environment [2], affecting aquatic wildlife forms and producing changes that threaten the sustainability of the ecosphere [3]. Continuous releases and chronic exposure to these chemicals not only can result in subtle effects on aquatic species but could also pose a risk to human health associated with consuming contaminated drinking water over a lifetime [4]. For instance, analgesic, anti-inflammatory, antibiotic, antiepileptic, antimicrobial, and antacid drugs are good examples of some of the most used PPCP types worldwide [5]. Nevertheless, due the huge number of PPCPs as well as the very low concentrations in which they usually occur in the environment, it is frequent to monitor particular anthropogenic chemical markers during pollution control programs. For instance, caffeine (CAF) and acetaminophen (ACF) are good examples of PPCPs tracers, since they are widely used anthropogenic drugs and the levels usually discharged from wastewaters into the environment are also very significant [3].

**Figure 1:**
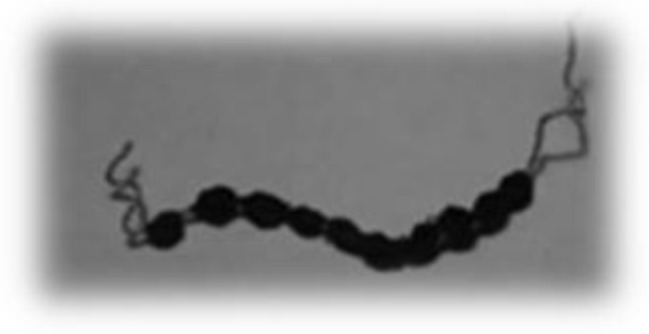
Image of device for multi-spheres adsorptive microextraction coated with several commercial activated carbons (MSAµE(AC)) used in the present work.

**Figure 2:**
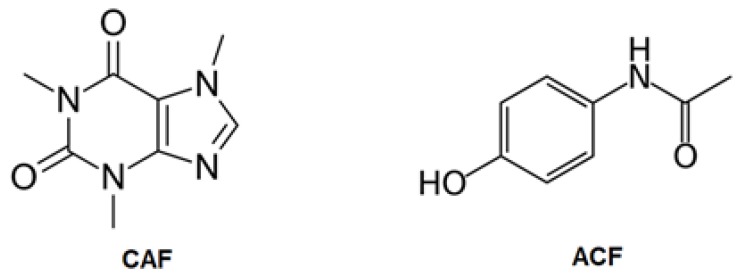
Chemical structures of the two markers of pharmaceuticals and personal care products PPCPs studied: caffeine (CAF) and acetaminophen (ACF).

Owing to the great environmental impact, it is mandatory to develop cheap, simple, rapid, and sensitive analytical strategies for monitoring trace levels of these anthropogenic chemical markers in the environment. State-of-the-art analytical methodologies for monitoring trace levels of PPCPs are usually based on sample enrichment techniques prior to chromatographic or hyphenated systems. In recent years, sample preparation methods are characterized by simplification and high throughput to enhance selectivity and sensitivity [6]. Therefore, for the determination of trace levels of PPCPs in environmental water matrices, solid-phase extraction (SPE) [3] and solid phase microextraction (SPME) [7] are well-established techniques that have showed great performance. Recently, adsorptive microextraction (AµE) techniques were introduced as a novel analytical approach that uses nanostructured sorbents and operates under the floating sampling technology [8,9]. For the implementation of these innovative static microextraction techniques, two configurations were originally designed, namely, analytical devices having bar or multi-spheres geometry. The present contribution proposes the application of a new analytical approach, namely multi-spheres adsorptive microextraction coated with several commercial activated carbons (MSAµE(AC), Figure 1) followed by high performance liquid chromatography with diode array detection (HPLC-DAD), for monitoring trace levels of two PPCPs markers (ACF and CAF, Figure 2) in environmental water matrices. According to previous exploratory data, MSAµE devices present much better stability compared to bar geometry devices when they are subjected to a more aggressive sample matrix because, in this case, thermal supporting promotes much higher robustness from the fixation point of view.

## 2. Results and Discussion

### 2.1. Characterization of the ACs

The commercial ACs used in this work were characterized by texture and surface chemistry (Table S1). From the pH_PZC_ values, AC(N) has basic surface and AC(R and N_OX_) have a slightly acidic surface. From the data depicted in Table S1, approximately 60% of the total volume of the ACs are constituted by microporous and 40% by mesoporous, which was in the same order of magnitude found in the literature [10].

### 2.2. HPLC-DAD Optimization

In this study, ACF and CAF were selected as anthropogenic tracer models. We evaluated the HPLC-DAD conditions, such as UV/vis spectra and chromatographic parameters. A wavelength (λ_max_) of 205 nm was selected, since it maximizes the DAD response for the target compounds. Using the chromatographic conditions described in Section 3.5, a good response was attained for the two tracers with suitable resolution and analytical time < 7 min. Instrumental sensitivity was also checked through the limits of detection (LODs) and quantification (LOQs) for both target analytes and calculated with a signal-to-noise ratio (S/N) of 3 and 10, respectively, resulting in LODs and LOQs of 120 and 400 µg L^−1^ for ACF, and 89 and 297 µg L^−1^ for CAF, respectively. Determination coefficients of 0.9999 were achieved with instrumental calibration having concentrations ranging from 0.6 to 10.0 mg L^−1^ (*n* = 6). Furthermore, instrumental precision was also assessed through repeated injections resulting in relative standard deviations (RSDs) lower than 8.2% and no carry-over was observed (<LODs).

### 2.3. Optimization of MSAµE(AC)-LD Assays

Several parameters affecting the microextraction and back-extraction stages efficiency of the MSAµE(AC)-LD approach, such as equilibrium time, agitation speed, matrix characteristics, and liquid desorption (LD) conditions, were tested and optimized [6].

**Figure 3:**
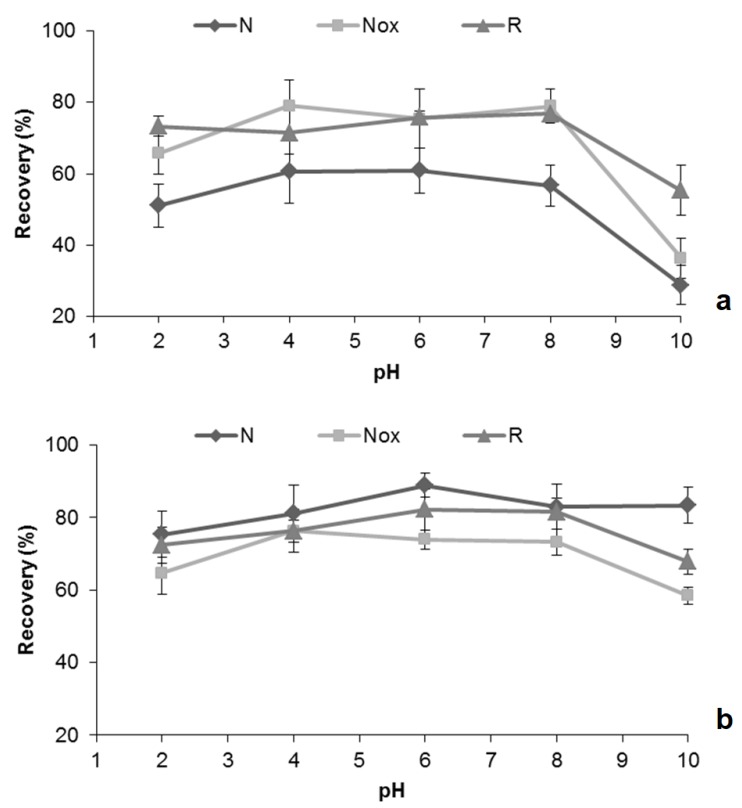
Effect of matrix pH on the recovery of ACF (**a**) and CAF (**b**) by MSAµE(AC) followed by liquid desorption and high-performance liquid chromatography with diode array detection (MSAµE(AC)-LD/HPLC-DAD), using different activated carbons (ACs) phases (R, N, and N_OX_).

In a first approach, we selected the most appropriate sorbent phase to microextract the target compounds. Since the pH of the extraction process plays an important role in the recovery yield, three ACs (R, N, and N_OX_) were assayed at five different pH values (2.0, 4.0, 6.0, 8.0, and 10.0), using standard conditions (microextraction: 17 h (1000 rpm) and back-extraction: formic acid (30 min)). This assessment is related with two factors, i.e., the analyte ionization and the charges on the ACs surface at a given pH. Therefore, the variation of pH affects the dissociation of the ACF and CAF molecules, as well as the surface chemistry of the sorbent involved due to the dissociation of the surface functional groups. The AC surface may be either positively or negatively charged depending on the nature of the AC. At a given pH, the AC surface and the analyte species may coexist in a complex system, in which similar or opposite charges may be present. However, as the ACs tested have distinct pH_PZC_ values (Table S1), the influence of the pH will be different. Figure 3 shows how the pH influences the recovery yield for each AC tested. For ACF (Figure 3a), the results show that to optimize the adsorption-desorption process, we must work in a pH between 4 and 8; once in this range, the difference observed was negligible, assuring that the ACF has not charged. The different performance of the three ACs was more pronounced with the surface chemistry than with the porous structure. In fact, N had the highest total, meso, and microporous volume (Table S1) despite its lower recovery yields. The ACs with more acidic characteristics (R and N_OX_) showed similar performance. Concerning CAF, AC(N_OX_) presented lower recovery and AC(N) had the maximum recovery value at pH 6.0. The lower recovery was reached at pH 10.0 and this decrease was more pronounced for ACF. This can be explained through the p*K*_a_ values, where ACF (p*K*_a_ 9.38) should present a negative charge at pH 10.0, while for CAF the p*K*_a_ is 10.40, which is higher than 10.0. AC(N) presented a different behavior with CAF and the recovery did not decrease at pH 10.0. In short, we can state that the AC(R) is the sorbent that allows a higher performance to simultaneously microextract both probe molecules.

The LD conditions that ensure complete back-extraction for the two tracers from the devices were also optimized. Formic acid and MeOH were tested to assess the LD performance. The results obtained showed that formic acid have better desorption capacity. Subsequently, the back-extraction time was also evaluated for both tracers by testing 15, 30, and 45 min of sonification time, from which 30 min seemed long enough without observing any carry-over effect. The evaporation step is essential for solvent switch, being necessary to carefully check for possible analyte losses during this process. For such purpose, three concentration levels were evaluated, where no significant losses were observed.

**Figure 4:**
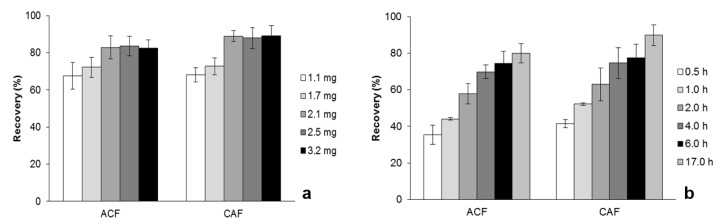
Effect of AC(R) mass (**a**) and equilibrium time (**b**) on the recovery of ACF and CAF by MSAµE(AC(R)-LD/HPLC-DAD.

For MSAµE(AC(R)) the number of multi-spheres can be chosen according to the expected content level for each target analyte. Consequently, five AC(R) masses (1.1, 1.7, 2.1, 2.5, and 3.2 mg) were tested. The data showed that 2.1 mg is enough to extract the target compounds at trace level (Figure 4a). According to the sorptive-based theory [11], extraction time and agitation speed are important parameters to better control the recovery conditions; during the extraction process, the multi-sphere devices are floating under the vortex created by the Teflon stir bar. We started with stirring rates of 750, 1000, and 1250 rpm, and the results obtained were negligible (data not shown). Subsequently, the equilibrium time was optimized (1000 rpm) by performing assays at 0.5, 1, 2, 4, 6, and 17 h, at room temperature. Figure 4b depicts the data obtained, where it can be observed that the recovery yields increase up to 17 h of extraction. Although slow kinetics are noticed, we decided to fix this parameter for further experiments, since this methodology can be performed overnight without any special requirements.

In accordance with previous works [10,12], the characteristics of the aqueous medium, i.e., ionic strength and polarity characteristics, are also important parameters with substantial effects on extraction efficiency. The ionic strength and polarity were modified through the addition of NaCl and MeOH (5, 10, and 15%) onto matrix media. Furthermore, the addition of MeOH reduced the recovery yield, with higher performance for ACF (Figure S1a). On the other hand, the ionic strength increment produced a very slight decrease on the recovery (Figure S1b).

### 2.4. Validation of the MSAμE(AC(R))-LD/HPLC-DAD Method

To obtain the recovery yields under optimized experimental conditions (microextraction stage: 2.1 mg of AC(R), 17 h (1000 rpm, pH 7.0); LD: formic acid (30 min)), assays were performed on ultra-pure water samples spiked at 10 µg L^−1^. The recovery yields achieved were higher than 75.0% (Table 1), indicating that both tracers have a high affinity to the selected AC.

**Figure 5:**
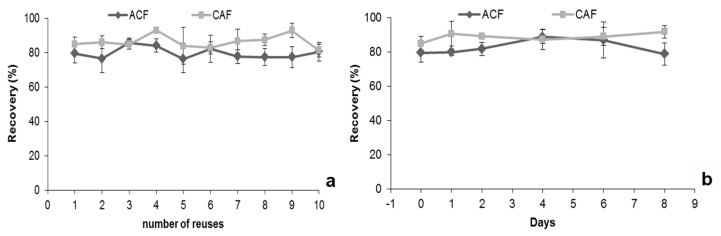
Comparison of recoveries of ACF and CAF after several reuses (**a**) and influence of storage time (**b**).

The lifetime of the microextraction device is also a very important parameter for practical utilization. The coating can be damaged by acidic and alkaline solutions as well as organic solvents. Therefore, the extraction capacity was evaluated after immersing the microextraction device in MeOH, *n*-hexane, 0.1 M HCl, and 0.1 M NaOH. From the data obtained (Figure S2), CAF-extraction ability showed no obvious decline after the microextraction devices were tested in different solvents for 48 h. For ACF, the recovery slightly decreased for the microextraction devices dipped in *n*-hexane. After each application, the microextraction devices can be reused and the number of possible uses dependents on the matrix type. After 10 applications of ACF and CAF using ultra-pure water matrix, the data indicate no obvious difference in efficiency (Figure 5a). It was also demonstrated that the microextraction devices can be stored with the analytes at 4 °C for 8 days without solute degradation (Figure 5b). This characteristic opens interesting features for on-site microextraction sampling, allowing the shipment of loaded devices to the laboratory after enrichment, instead of samples.

To validate the proposed methodology, parameters such as linearity, LOD, LOQ, and precision were evaluated. For linearity, the determination coefficients (*r*^2^) were higher than 0.9976 with concentration levels ranging from 4.0 to 40.0 µg L^−1^ (*n* = 5). For LODs and LOQs, values of 0.8–1.2 µg L^−1^ and 2.8–4.0 µg L^−1^ were achieved, respectively. Precision was evaluated using within and between-day repeatability with three concentrations levels (4.0, 20.0, and 40.0 µg L^−1^, *n* = 6) and expressed as relative standard deviation (RSD = 13.8%). According to the requirements of Directive 98/83/EC [13], a value under 25% for organic compounds in water matrices may be considered acceptable.

Finally, we compared the average recovery yields of the proposed methodology with those of other microextraction techniques already established (Table 1). As can be observed, the proposed methodology revealed recovery levels for ACF almost two-fold better and of the same order of magnitude for CAF when compared with other well-established microextraction techniques. It must also be emphasized that the proposed methodology uses small amounts of sample during the extraction stage and less organic solvent during the back-extraction stage than many other microextraction approaches reported in the literature (e.g., SPE) [14,15,16,17,18].

### 2.5. Application to Environmental Water Matrices

**Figure 6:**
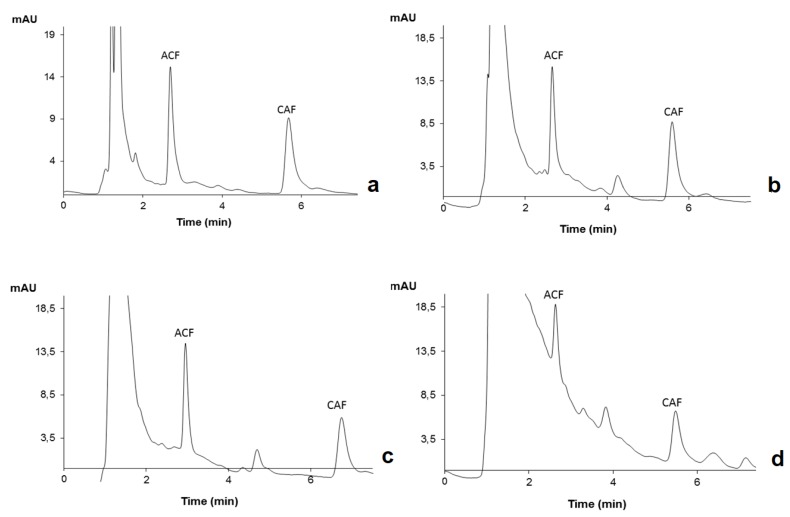
Chromatograms obtained from assays performed on ultra-pure water (**a**), superficial water (**b**), sea water (**c**), and urban wastewater (**d**) by MSAµE(AC(R))-LD/HPLC-DAD under optimized experimental conditions.

The efficiency of sample preparation can be affected by the complexity of the matrix involved. For instance, substantial levels of dissolved or suspended inorganic or organic matter contained in water matrices may interfere with the extraction process of the target compounds; therefore, the yields might drastically change from sample to sample [19]. In this work, the standard addition method (SAM) was used to minimize the occurrence of matrix effects. Table 2 summarizes the data obtained by SAM assays on the different matrices studied under optimized experimental conditions. When comparing the responses obtained when AC(R) is used in ultra-pure, surface, sea, and treated wastewater matrices, a very slow decrease in the signal for target compounds is noticed. The matrix effects were evident in the treated wastewater samples, thus justifying the quantification performed by the SAM in this kind of sample for this type of target compounds. Figure 6 exemplifies chromatograms obtained from assays performed on spiked (10 µg L^−1^) ultra-pure (a), surface (b), sea (c), and urban wastewater (d) samples by MSAµE(AC(R))-LD/HPLC-DAD under optimized experimental conditions, where good sensitivity and remarkable selectivity are noticed.

In short, it must be emphasized that the present methodology is easy to work-up and environmentally friendly, and requires low sample and solvent volumes. Therefore, this new methodology presents advantages in relation to other well established microextraction approaches [20].

## 3. Materials and Methods

### 3.1. Samples and Reagents

HPLC-grade methanol (MeOH, 99.9%), formic acid (99%) and *n*-hexane (96%) were purchased from Merck (Darmstadt, Germany). Ultra-pure water was obtained from Milli-Q water purification systems (Millipore, Burlington, MA, USA). Sodium chloride (NaCl, 99.9%) was supplied by José M.G. dos Santos (Lisbon, Portugal). Hydrochloric acid (HCl, 37%) and phosphoric acid (85%) were obtained from Riedel-de Haën (Seelze, Germany). Sodium hydroxide (NaOH, 98.0%) was obtained from AnalaR BDH Chemicals (London, UK). ACF (98.0%) and CAF (99.0%) were supplied from Sigma Aldrich (Darmstadt, Germany). A stock solution of each compound (200 mg L^−1^) was prepared in MeOH and stored refrigerated at −20 °C. An intermediate standard solution containing both compounds at a concentration of 10 mg L^−1^ was obtained after mixing individual stock solutions, diluting with MeOH/water (25/75%) and stored at 4 °C. The commercial AC(N) was supplied by Salmon & Cia. (Lisbon, Portugal) and the AC(R) from Riedel-de Haën (Seelze, Germany). The AC(N_OX_) was obtained by oxidized AC(N) with nitric acid (HNO_3_, 20%). All environmental samples were collected in Portugal previously filtered and stored refrigerated at 4 °C until their analysis. Discrete wastewater samples were collected from the effluent of an urban treatment plant located at Cartaxo after all wastewater treatment; surface water samples were collected from Tagus river bank at Lisbon (Oriente); sea water samples were collected in Algarve seaside.

### 3.2. ACs Characterization

The procedure of textural and surface chemistry characterization of all ACs (N, N_OX_, and R) was performed as described by Mestre et al. [21].

### 3.3. MSAµE Devices Preparation

The microextraction devices were lab-made (Figure 1) as previously reported [9]. Before use, the microextraction devices were cleaned through a sonification treatment using MeOH and ultra-pure water.

### 3.4. Recovery Assays and Method Validation

Typical assays were performed with 30 mL of ultra-pure water spiked with a standard mixture at the desired concentration, followed by the introduction of the microextraction device and a conventional Teflon magnetic bar.

Several parameters of MSAµE(AC), such as coating phase (N, N_OX_ or R), sorbent amount (1.1, 1.7, 2.1, 2.5, and 3.2 mg), equilibrium time (0.5, 1, 2, 4, 6, and 17 h), agitation speed (750, 1000, and 1250 rpm), pH (2, 4, 6, 8, and 10), organic modifier (MeOH; 5, 10, and 15%, *v/v*), ionic strength (NaCl; 5, 10, and 15%, *w/v*), desorption solvent (MeOH and formic acid), and desorption time (15, 30, and 45 min) were systematically studied and optimized (*n* = 3). After back-extraction, the devices were removed, and the stripping solvent was evaporated to dryness under purified nitrogen (>99.5%). The residues were redissolved in 200 µL of MeOH/water (25/75%) and afterwards, the vials were closed with a seal and placed on the auto-sampler for HPLC-DAD analysis. Blank assays were also carried out without spiking. More detail can be found in our previous reports [6,10,12]. For testing chemical and mechanical stability, the microextraction devices were immersed in vials loaded with MeOH, *n*-hexane, HCl (0.1 M), and NaOH (0.1 M) solutions at room temperature. After 48 h, the microextraction devices were taken out for the extraction assay. The analyte recoveries (10 µg L^−1^) before and after dipping in different solutions were compared. The SAM was used for quantification on real matrices, and 30 mL of wastewater, sea, and river water samples were used. To guarantee maximum control of the analytical methodology, the samples were first and foremost fortified with four working standards to produce the corresponding spiking levels for the target compounds (4–40 µg L^−1^) and blank assays (“zero-point”) were also performed without spiking. All assays were done in triplicate.

### 3.5. HPLC-DAD Settings

An Agilent 1100 Series LC system (Agilent Technologies, Waldbronn, Germany) equipped with a C18 column (Tracer excel, 150 mm × 4.0 mm, 5 µm particle size, Teknokroma, Barcelona, Spain) and LC3D ChemStation software (version Rev.A.10.02[1757], Waldbronn, Germany) was used for the HPLC-DAD analysis. The mobile phase consisted in a mixture of 25% (*v/v*) MeOH solution and 0.1% phosphoric acid aqueous solution (flow: 1 mL min^−1^). The column temperature was maintained at 25 °C, the injection volume was 20 µL with a draw speed of 200 µL min^−1^ and the detector was set at 205 nm.

## 4. Conclusions

The MSAµE(AC(R))-LD/HPLC-DAD methodology proposed in this work offers a new practical alternative method for analysis of PPCPs tracers such as CAF and ACF in environmental water samples. Under optimized experimental conditions, good analytical performance was attained as well as suitable detection limits and linear dynamic ranges. The textural and surface properties of the ACs were correlated with the analytical results for a better understanding of the overall process. By using the SAM, the established methodology showed a good response for the analysis of ACF and CAF in water samples such as surface, sea, and wastewater matrices. Furthermore, MSAµE(AC) is cost-effective and easy to work-up, demonstrating to be a remarkable analytical tool for trace analysis of priority and emerging pollutants in environmental water matrices. The novel methodology proposed herein is compliant with green analytical chemistry principles and a good alternative to overcome the limitations of other technologies for trace analysis of polar compounds.

## Figures and Tables

**Table 1:** Comparison of average recovery yields of both tracers achieved by the current method and other microextraction techniques.

Compounds	Microextraction Techniques	Recovery (%)	Refs.
CAF	MSAµE	75.3	This work
	SPE	99.3	13
	SPE	88.0	14
	SPME	98.5	15
ACF	MSAµE	82.6	This work
	SPE	11.3	16
	SPE	47.0	17

**Table 2:** Regression parameters obtained with the standard addition method (SAM) and average recovery yields for ACF and CAF in ultra-pure, surface, sea, and wastewater matrices obtained by MSAµE(AC(R))-LD/HPLC-DAD under optimized experimental conditions.

Compounds		Recovery (%);		
		(*r*^2^)		
	Ultra-Pure Water	Surface Water	Sea Water	Wastewater
ACF	75.3 ± 9.5	64.8 ± 8.3	73.1 ± 2.9	50.1 ± 2.2
	(0.9983)	(0.9960)	(0.9970)	(0.9953)
CAF	82.6 ± 2.9	83.5 ± 1.1	74.6 ± 2.7	54.7 ± 5.6
	(0.9976)	(0.9935)	(0.9975)	(0.9989)

## Supplementary Materials

The following are available online. Table S1. Nanotextural and chemical characteristics of the commercial ACs. Figure S1. Effect of matrix polarity (a) and ionic strength (b) on the recovery of ACF and CAF by MSAµE(AC(R)-LD/HPLC-DAD. Figure S2. Evaluation of extraction capacity after immersing the devices in different solvents.
