# Supplementary material for: Multi-Spheres Adsorptive Microextraction (MSAμE)—Application of a Novel Analytical Approach for Monitoring Chemical Anthropogenic Markers in Environmental Water Matrices
Source: Molecules. 2019 Mar 7;24(5):931. doi: 10.3390/molecules24050931 (PMC6429196; doi:10.3390/molecules24050931)
Supplement: Supplementary file 1 [file molecules-24-00931-s001.pdf]

Supplementary data

Table S1. Nanotextural and chemical characteristics of the commercial ACs.

AC	pH_{PZC}	A_{BET}* $cm^3\ g^{-1}$	V_{total} [a] $cm^3\ g^{-1}$	V_{meso} [b] $cm^3\ g^{-1}$	α_s method: $V_{\alpha\ total}$ $cm^3\ g^{-1}$	α_s method: $V_{\alpha\ ultra}$ $cm^3\ g^{-1}$	α_s method: $V_{\alpha\ super}$ $cm^3\ g^{-1}$
R	6.5	937	0.65	0.36	0.29	0.10	0.19
N	8.4	1065	0.70	0.30	0.40	0.02	0.38
N_{OX}	5.5	875	0.51	0.14	0.37	0.00	0.37

[a] Volume adsorbed at $p/p^0 = 0.95$; [b] Difference between V_{total} and $V_{\alpha\ total}$.
* Specific surface area obtained by BET equation.

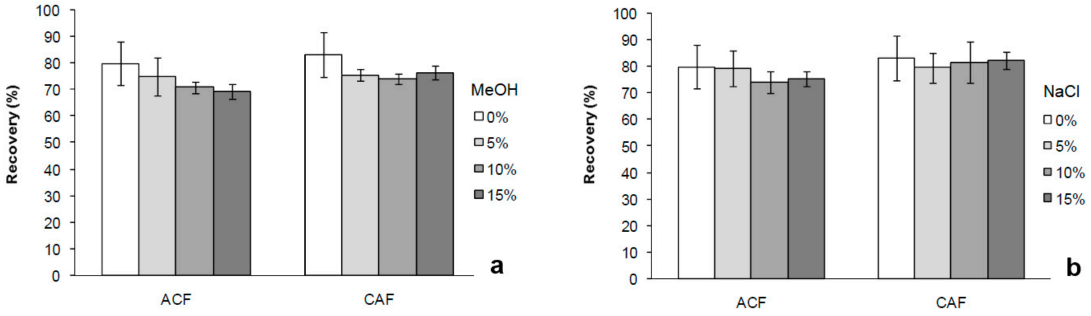

Figure S1. Effect of matrix polarity (a) and ionic strength (b) on the recovery of ACF and CAF by MSAμE(AC(R)-LD/HPLC-DAD.

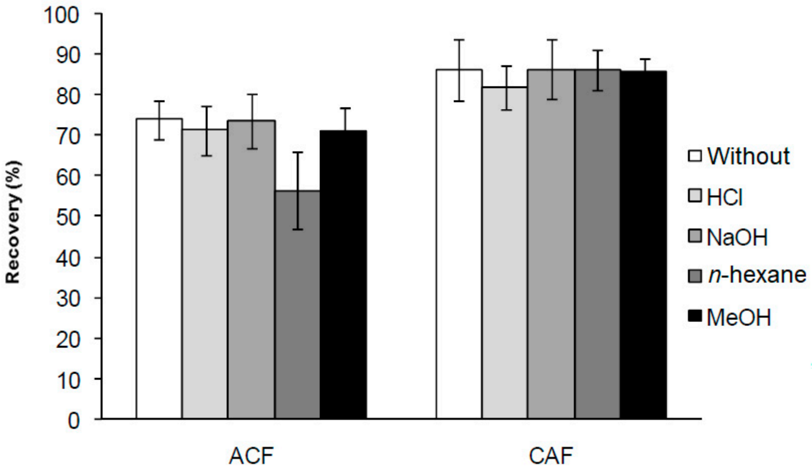

Figure S2. Evaluation of extraction capacity after immersing the devices in different solvents.
